# Effect of coaches’ interpersonal style on young athletes’ individual resilience and team adherence intention: a season-long investigation

**DOI:** 10.1186/s40359-023-01445-3

**Published:** 2023-11-25

**Authors:** Rubén Llanos-Muñoz, Juan J. Pulido, Hadi Nobari, Javier Raya-González, Miguel A. López-Gajardo

**Affiliations:** 1 Faculty of Teacher Training, Department of Didactics of Musical, Plastic and Corporal Expression, University of Extremadura, Cáceres, Spain; 2 Faculty of Education and Psychology, Department of Didactics of Musical, Plastic and Corporal Expression, University of Extremadura, Badajoz, Spain; 3 Faculty of Educational Sciences and Psychology, Departament of Exercise Physiology, Universitiy of Mohaghegh Ardabili, Ardabil, 56199-11367 Iran; 4 Faculty of Sports Science, Department of Didactics of Musical, Plastic and Corporal Expression, University of Extremadura, Cáceres, Spain

**Keywords:** Resilience, Interpersonal coaching style, Sport performance, Internal psychological load

## Abstract

**Background:**

In the sports context, coaches must be able to improve their players physically, psychologically, and socially. Hence, a fundamental part of this process is the athlete’s individual resilience (IR).

**Methods:**

Three hundred and fifteen youth team-sport players (boys: *n* = 283; *M*_age_ = 16.02, *SD* = 0.56; and girls: *n* = 32; *M*_age_ = 15.92, *SD* = 0.62) completed the measures of coach’s interpersonal style, individual resilience, perceived performance, and team adherence intention (intention to remain on the same team the following year) twice (Time 1: mid-season; Time 2: end-season). Structural equation modeling was used to test the relationships between variables.

**Results:**

The results showed that coach support was positively related to IR (*p* < 0.001) and, in turn, IR to individual (*p* < 0.01) and team performance (*p* < 0.05) at Time 1, and to individual performance (*p* < 0.001) and team adherence intention at Time 2 (*p* < 0.01). In addition, team performance at Time 2 was positively related to team adherence intention (*p* < 0.001). Finally, a mediating effect of IR was observed between interpersonal coaching style, individual and team performance, and team adherence intention.

**Conclusions:**

These results show the importance of a supportive interpersonal coaching style to foster athletes’ levels of resilience, which could have positive consequences in performance (individual and team) and team adherence intention.

## Introduction

An individual’s personality is a component of the psychosocial aspects essential to improve sports performance [1]. According to Ramirez-Granizo et al. [2], sports practice is considered a protective factor against stress or anxiety, as it promotes individuals’ intrapersonal knowledge, discovering their weaknesses and strengths, which improves their internal psychological load. In terms of individual factors, people who have a flexible and balanced personality, capable of affective and physiological responses to environmental circumstances, are the most likely to develop resilience [3].

Accordingly, Sarkar and Fletcher [4] state that athletes’ development of individual resilience (IR) allows them to overcome and move forward in the presence of stressful and anxiogenic events, as well as to consider these situations an opportunity for personal growth to expand their capabilities and master the situation through motivation, instead of perceiving risk and adversity as threats. Resilience has been studied over the decades, and the most recent and comprehensive definition considers resilience as “the role of mental processes and behavior in promoting personal assets and protecting an individual from the potential negative effect of stressors” [5]. Consequently, it is considered a capacity that depends on the individual and how they interact with their context. We note that situations for developing resilience must not always be adverse but can require the individual to adapt positively to the new demands of the environment (e.g., promotion to a higher league).

In the sports context, coaches often use resilience to describe athletes’ or teams’ favorable responses to different situations [6]. Early research in sports settings focused on establishing the relationship between resilience and performance failure [7–9]. Research by Krane and Williams or Gould and Maynard [10, 11] found that better stress and pressure management -coping with anxiogenic situations in training and competition- leads to higher levels of sports performance. Subsequently, Galli and Vealey [6] developed a conceptual model of resilience in sports. They defined and asserted that adversity (e.g., injury), sociocultural influence (e.g., coach and family support), and personal resources (e.g., determination) are critical components in the resilience process and, depending on the directionality of these variables, can lead to either positive or negative learning and/or performance outcomes. However, the model presents several drawbacks [12]. Thus, to address the shortcomings of this model, [13] subsequently developed a theory of psychological resilience and optimal sports performance. Social support is one of the psychological factors determining the individual’s challenge and metacognition within this theory. This is observed from the perception of the support of different agents involved in the athlete’s training and competition process (e.g., family, coach, and peer support). The stress-resilience-performance relationship is strengthened as a function of the interaction and perception of the support available from the various agents. This finding, along with previous research [14, 15], shows that social support can buffer the effects of stress and is one of the crucial factors of resilience in elite sports. Therefore, it is necessary to determine whether the stress-resilience-performance relationship exists in training categories.

Considering a team sport setting, the present research takes the interpersonal coaching style (i.e., need-support and need-thwarting) as the central axis. The coaches’ role is crucial because they can establish the appropriate strategies for athletes to deal with stressors positively and, consequently, increase their self-esteem, self-concept, and well-being, achieving a resilient profile [16]. Based on the Self-Determination Theory (SDT), which postulates the basic psychological needs (BPN) (autonomy, competence, and relatedness) that allow people’s personal development and growth, it is suggested that the coach can influence athletes through two very different interpersonal styles: need-supportive and/or need-thwarting behaviors [17]. When the coaches support their athletes (i.e., taking on the others’ perspective, being understandable and flexible, motivating through interest, and justifying why they ask for certain things), they favor the satisfaction of the BPN, but when coaches adopt a thwarting style (i.e., behaving coercively, pressuring their athletes, being authoritarian to impose their way of thinking and behaving), they frustrate the BPN [17]. That is why the coach’s role, from the duality of support and thwarting -in addition to influencing the athlete’s motivation- can also help athletes develop IR, perform optimally, and express team adherence intention if an adequate motivational climate is created during training [18].

Based on previous studies, White and Bennie [19] investigated the development of resilience in gymnastics through the perceptions of gymnasts and coaches. Their results show that positive sports environment and interpersonal relationships in youth sports are appropriate for developing resilience. In this line, Trigueros et al. [20] demonstrated that when the coach fosters a relationship based on the athlete’s autonomy, the athlete experiences a much deeper and more rewarding learning process when facing adverse situations. Similarly, athletes are more likely to evaluate stressors positively if the coach helps them develop that optimal level of IR for their progress through adequate resources and social support [21]. Research has corroborated the development of resilience in the sports context. Fletcher and Sarkar [13] sought to explain the relationship between psychological IR and sports performance in 12 Olympic gold medallists. Their results revealed numerous factors, such as personality, motivation, or social support, that facilitate responses preceding optimal sports performance. Furthermore, various studies argue that athletes’ resilience is positively related to perceived optimism and negatively associated with stress and burnout [12, 22, 23]. In this regard, the coach’s interaction with their athletes will determine how athletes respond to the stressors they meet [13]. There is growing evidence that resilience is an essential psychological phenomenon for achieving high levels of sports performance (see, e.g., [18, 24]).

In turn, according to the existing literature, the relationship between IR and team adherence intention has been found in other fields such as hospitality [25], education [26], or work [27]. However, no evidence in the field of sports corroborates this relationship, so the results obtained in this work are relevant to understanding whether the development of this ability favors adherence to sports practice by athletes in training sports. Regarding adherence to sports practice, Almagro et al. [28] showed that sports performance is directly related to team adherence intention the following season. Advancing in knowledge of team psychology in competitive sports provides insight into the processes that underpin the collective functioning that precedes optimal team performance in a dynamic and competitive environment [29]. Previous studies have corroborated the mediating effect of resilience on basic psychological needs [30], anxiety in the sports context [31], and self-efficacy [32]. Nevertheless, the body of knowledge in this area requires more knowledge and the opportunity to elucidate the athlete-team-coach relationship.

**Fig. 1:**
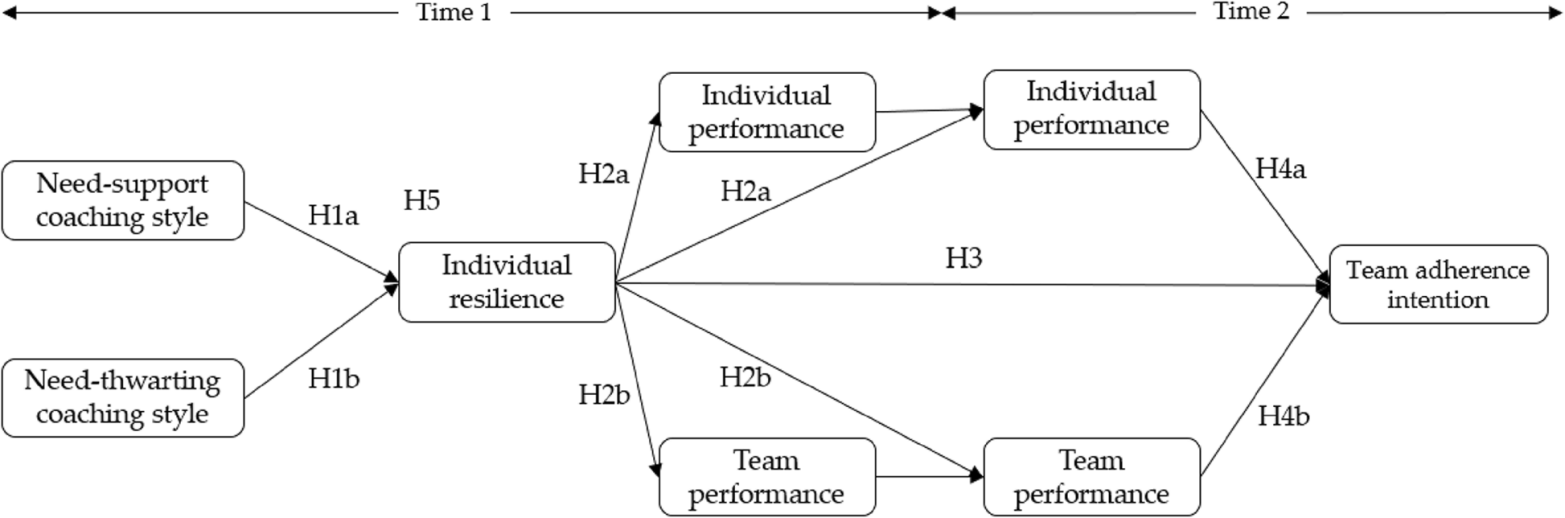
Hypothetical model of the relationships between need-support and need-thwarting coaching styles, individual resilience, players’ perceived performance (individual and team), and players’ intentions to persist in the team

The present study offers a new possibility for research in training sports, given that there is little scientific information on resilience in adolescent training categories of team sports. Based on the established theoretical framework, it is necessary to develop a model to observe the effect of need-support and need-thwarting interpersonal coaching style on IR and how the development of IR can have consequences on both individual and team performance, as well as on the team players’ team adherence intention over some time (see Fig. 1). Therefore, taking into account the findings of previous studies, the present study has the following aims: (1) to analyze how need-support and need-thwarting coaching behaviors are related to players’ IR; (2) to analyze the relationship between IR and individual and team performance as perceived by players at Times 1 and 2; (3) to test the relationship between individual resilience and team adherence intention the following season; (4) to test the association between individual and team performance at Time 2 and team adherence intention the following season; (5) to analyze whether IR plays a positive and indirect role between need-support and need-thwarting interpersonal coaching styles and the players’ perceived individual and team performance at both times, and team adherence intention the following season. Based on these objectives, we hypothesize that coach need-supportive behavior (H_1a_) will be positively related to athletes’ IR, whereas coach need-thwarting style (H_1b_) will be negatively related. Secondly, IR will be positively associated with players’ perceived individual (H_2a_) and team (H_2b_) performance at Time 1 and Time 2. Thirdly, (H_3_), IR will have a positive relationship with team adherence intention the following season. Fourthly, individual (H_4a_) and team (H_4b_) performance at Time 2 will be positively related to team adherence intention the following season. Finally, IR will act positively and indirectly between coach need-supportive and need-thwarting behaviors and individual (H_5a_) and team (H_5b_) performance, as well as team adherence intention the following season (H_5c_).

## Materials and methods

### Participants

The participants were selected purposefully and based on the inclusion criteria of this research. The study inclusion criteria were: (i) being part of the team that participates and competes in the regional categories, either football or volleyball; (ii) completing the consent form signed by the parents and/or legal guardians of minors; and (iii) correctly completing the questionnaires at both times. Finally, 315 athletes (*M*_*age*_ = 15.72, *SD* = 1.33) corresponding to 34 football and volleyball teams (31 male and 3 female) from Extremadura (Spain) participated in the present study. Of the participants, 283 were boys (*M*_*age*_ = 16.02, *SD* = 0.56), and 32 were girls (*M*_*age*_ = 15.92, *SD* = 0.62). All of them were U-16 (*n* = 158) and U-18 of Spain (*n* = 157) and competed during the 2021/2022 season.

### Procedures

The participants were treated following the ethical guidelines of the American Psychological Association [33] regarding consent, confidentiality, and anonymity of responses. The study received ethical approval from the University. The principal investigator contacted all team leaders to clarify the study’s objectives and request their participation in the project. In addition, as the athletes were minors, informed consent was obtained and signed by the athletes and a parent and/or legal guardian, recording their voluntary participation in the present research. Regarding data collection, the present study was a cross-sectional design with variables measured at different times [34]. Thus, data were collected at two time periods separated by 4 months: Time 1 and Time 2. At Time 1, athletes completed the mid-season of the coach’s interpersonal style questionnaire (i.e., support and control), IR, and perceived individual and team performance. At Time 2, they completed the individual and perceived team performance and adherence questionnaires. Following receipt of the signed consent form, the principal investigator provided all athletes and coaches with a link to a Google website to complete the questionnaires online before a training session,. The questionnaires were anonymous, and at no time were the data processed in a personal way. However, the principal investigator was present during the data collection, supervising the whole process and answering specific questions if necessary. The questionnaires were completed individually, without distractions, and for approximately 8–12 min. To compare the results at both times for the same players, an ID was associated with each player.

### Material and testing

#### Coach’s interpersonal style

An adaptation of the Teaching Interpersonal Style Questionnaire in Physical Education (CISQ), developed by Pulido et al. [35], was used to assess coach interpersonal style as perceived by the young athletes. This questionnaire starts with this stem sentence: “During training sessions, our coach...” followed by 22 items corresponding to two main factors (11 for coach support and 11 for coach thwarting). Specifically, four items were included to measure the perception of autonomy support (e.g., “...tries to give us options when performing exercises”), three items assessed competence support (e.g., “...favors learning and improving knowledge”), and three items reflected relatedness support (e.g., “...encourages good peer relationships at all times”). On the other hand, a set of four items measured perceived autonomy-thwarting (e.g., “...requires me to do things in a certain way”), three items assessed competence-thwarting (e.g., “...sets up situations that make me feel unable to perform”), and three items reflected relatedness-thwarting (e.g., “...sometimes shows rejection towards me “). Responses were rated on a 5-point Likert scale ranging from 1 (*strongly disagree*) to 5 (*strongly agree*). Hierarchical confirmatory factor analysis (H-CFA) showed an adequate fit to the data: χ^2^ = 353.619, *df* = 179, *p* < 0.001, CFI = 0.91, TLI = 0.90, RMSEA = 0.06, 95% CI [0.047, 0.064], SRMR = 0.06. Furthermore, the internal consistency showed optimal values for the need-supportive coaching style factor (α = 0.81; ω = 0.80) and need-thwarting coaching style factor (α = 0.91; ω = 0.91).

#### Individual resilience

To assess athletes’ resilience, the present study used the Connor-Davidson Resilience Scale (CD-RISC [36];) in its shortened version of 10 items validated by Campbell-Sills and Stein [37] (CD-RISC10). The scale begins with the phrase, “Please indicate the extent to which you agree with the following statements or events that have occurred in the past month...”, followed by a total of 10 items (e.g., “the capacity to adapt to change”), which are scored on a 5-point Likert scale, ranging from 0 (*not at all*) to 4 (*almost always*). Confirmatory factor analysis (CFA) showed an adequate model fit to the data: χ^2^ = 32.966, *df* = 24, *p* < 0.001, CFI = 0.98, TLI = 0.96, RMSEA = 0.03, 95% CI [0.00, 0.06], SRMR = 0.03. Furthermore, the general dimension showed adequate levels of internal consistency (α = 0.79; ω = 0.79).

#### Perceived individual and team performance

In the absence of a standardized and validated instrument to analyze sports performance due to the high number of interactions in a competition, some researchers have used player perceptions/ratings to estimate performance [38, 39]. This appears to be an ecologically valid and reliable way of assessing performance in team sports [40]. The scale previously used by Dithurbide et al. [41], consisting of a single item, was used. Players were asked to rate their team’s performance during the season with this item (e.g., “the team’s performance during the season has been...”). This item was also adapted to measure each player’s perceived performance (e.g., “your individual performance during the season was...”). Both items were followed by a 10-point Likert scale, ranging from 1 (*poor*) to 10 (*excellent*).

#### Team adherence intention

The athletes’ team adherence intention the following season was measured through three items translated into Spanish and used to assess this intention: “Would you like to continue next year ... (1) on the same team? (2) ... with the same coach? and (3) ... with the same teammates?” This question has been used in previous research [42–45]. A 10-point Likert scale ranging from 1 (*strongly disagree*) to 10 (*strongly agree*) was used to evaluate the responses. Also, a general factor demonstrated high levels of internal consistency (α = 0.88; ω = 0.88).

#### Statistical analysis

The statistical program Mplus, version 7.3 [46], was used for data analysis. First, CFA was performed as a preliminary analysis on each scale to determine the model’s fit. Second, descriptive statistics, bivariate correlations, and reliability analyses were performed. Third, in the primary analyses, due to the small sample size for the between-level variable (i.e., eight teams), we only tested a model targeting the individual level of analysis. Therefore, a structural equation model (SEM) was completed to test the model hypothesized in this study, using the Mplus COMPLEX instruction to control for the nesting of players within teams, and a multiple linear regression (MLR estimator [47]). Finally, indirect effects were tested using the bias-corrected bootstrap method (10,000 samples with 95% confidence corrected for bias intervals -IC- [48]) with the maximum likelihood procedure (ML; bootstrapping is unavailable when using MLR estimation). This method is the most effective way to identify indirect relationships when their theoretical distributions are asymmetric [48]. This method does not require the assumptions and preliminary checks for a mediation analysis [49]. Lastly, to assess the models’ fit, we used the following indices: chi-square (χ^2^), comparative fit index (CFI), Tucker Lewis index (TLI), root mean square error of approximation (RMSEA), and standardized root mean square residual (SRMR). According to Schumacker and Lomax [50], incremental indexes (CFI and TLI) indicate acceptable fit when values of .90 or higher are obtained. Regarding RMSEA and SRMR, .08 or .06 have been established as acceptable cut-off points [51]. Lastly, optimal chi-square and degrees of freedom (*df*) values should be non-significant, but significant values do not necessarily indicate poor model fit if all other values are appropriate [50].

## Results 

### Descriptive statistics and Cronbach’s alpha coefficients

Regarding the descriptive analysis of the present work, Table 1 shows the means, standard deviations, reliability analysis, and bivariate correlations of the variables that comprised the model. All the scales had acceptable internal consistency (α > 0.70; [52] ω > 0.70; [53]).

**Table 1:** Means, standard deviations, bivariate correlations, and reliability analysis of the variables

Variable	*M*	*SD*	*α*	*ω*	1	2	3	4	5	6	7	8
NS-CS	4.22	0.51	0.81	0.80	–							
NT-CS	1.86	0.82	0.91	0.91	−0.37^***^	–						
IR	3.29	0.52	0.79	0.79	0.49^***^	−0.25^***^	–					
IPT1	7.63	1.81	–	–	0.17^**^	−0.12^*^	0.28^***^	–				
TPT1	7.85	1.69	–	–	0.14^**^	−0.05	0.12^*^	0.38^***^	–			
IPT2	7.44	1.80	–	–	0.21^***^	−0.12^*^	0.34^***^	0.43^***^	0.21^***^	–		
TPT2	7.88	1.69	–	–	0.21^***^	−0.11^*^	0.12^*^	0.21^***^	0.44^***^	0.47^***^	–	
ITA	8.38	2.00	0.88	0.88	0.33^***^	−0.27^***^	0.21^***^	0.04	0.18^***^	0.26^***^	0.45^***^	–

*p < 0.05; **p < 0.01; ***p < 0.001. *NS-CS* Need-supportive coaching style, *NT-CS* Need-thwarting coaching style, *IR* Individual Resilience, *IPT1* Individual Performance at Time 1, *TPT1* Team Performance at Time 1, *IPT2* Individual Performance at Time 2, *TPT2* Team Performance at Time 2, *ITA* Team Adherence Intention

Considering the coach’s need-supportive style, a positive relationship was observed with the IR developed by the athlete (*r* = 0.49, *p* < 0.001). The coach’s need-supportive style was also positively related to individual (*r* = 0.17, *p* < 0.01) and team (*r* = 0.14, *p* < 0.01) performance perceived at Time 1 and at Time 2 (*r* = 0.21, *p* < 0.001, and *r* = 0.21, *p* < 0.001, for individual and team performance, respectively). Finally, there was also a positive relationship between the coach’s need-supportive and team adherence intention the following season (*r* = 0.33, *p* < 0.001).

On the other hand, the coach’s need-thwarting behavior had a negative relationship with the athlete’s IR (*r* = − 0.25, *p* < 0.001). Concerning performance, the coach’s need-thwarting style negatively influenced individual performance perceived by the athlete (*r* = − 0.12, *p* < 0.05) at Time 1. At Time 2, the coach’s need-thwarting style was also negatively related to individual (*r* = − 0.12, *p* < 0.05) and team (*r* = − 0.11, *p* < 0.05) perceived performance. Furthermore, concerning team adherence intention, the coach’s need-thwarting style was negatively related to team adherence intention (*r* = − 0.27, *p* < 0.001).

The development of IR was positively related to individual (*r* = 0.28, *p* < 0.001) and team (*r* = 0.12, *p* < 0.05) performance at Time 1. Similarly, IR was positively related to individual (*r* = 0.34, *p* < 0.001) and team (*r* = 0.12, *p* < 0.05) performance at Time 2. The positive relationship between IR and team adherence intention (*r* = 0.21, *p* < 0.001) was also important. Finally, a positive relationship was found between perceived individual and team performance at Time 2 and team adherence intention on the same team (*r* = 0.26, *p* < 0.001 and *r* = 0.45, *p* < 0.001, respectively). Standard deviations, normality, and Cronbach’s alpha coefficients for each variable are presented in Table 1. Regarding means, participants generally obtained scores above the scales’ midpoint for authentic leadership, coaching competency, perceived justice, role ambiguity, cohesion, TMS, and collective efficacy. Participants also obtained scores below the scales’ midpoint for role conflict and team conflict.

### Main analysis

**Fig. 2:**
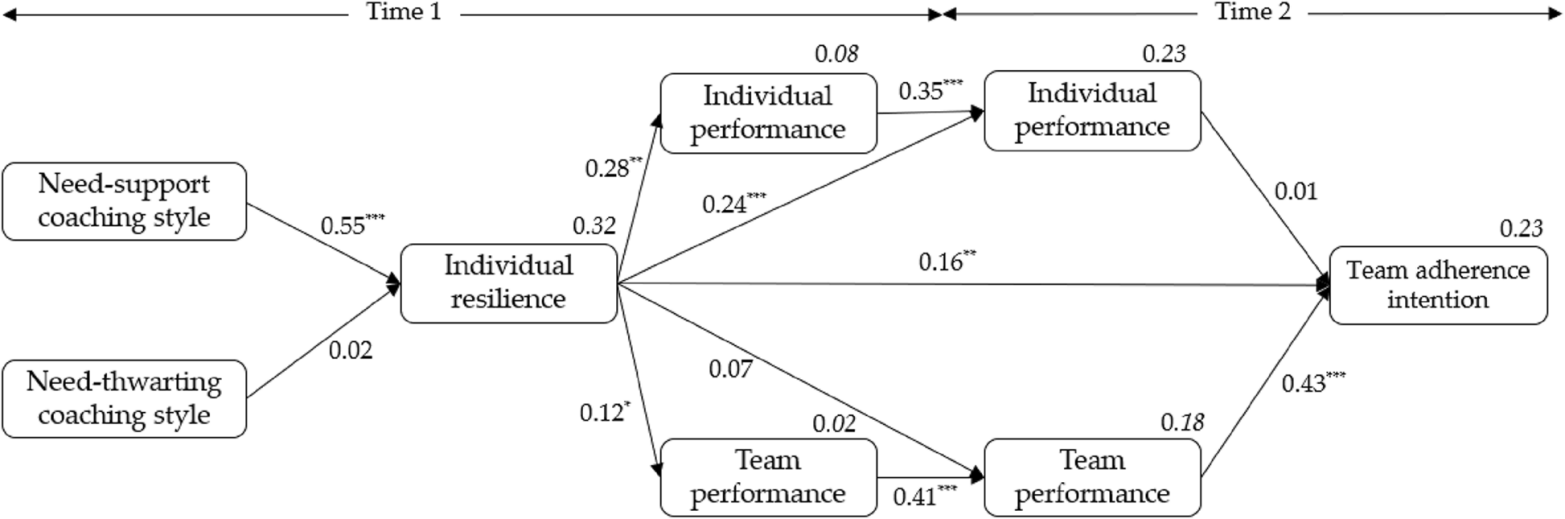
SEM of the relationship between perceived coach need-support and need-thwarting behaviors, individual resilience, individual and team performance, and team persistence. Note. *p < 0.05; **p < 0.01; ***p < 0.001

Figure 2 shows the SEM of the relationships established in the research objectives. The model showed the following fit data: χ^2^ = 114.62, *df* = 0.46, *p* < 0.001, CFI = 0.92, TLI = 0.89, RMSEA = 0.07, 95% CI [0.05, 0.08], SRMR = 0.07. To explain the results, we will follow the order established by the objectives and hypotheses (see Fig. 1) of the present work. Thus, concerning H_1_, the results showed that only the coach’s need-supportive style had a positive relationship with IR (β = 0.55, *p* < 0.001, 95% CI [0.35, 0.75]). Regarding H_2a_, IR had a positive association with individual performance (Time 1: β = 0.28, *p* < 0.01, 95% CI [0.11, 0.44]; Time 2: β = 0.24, *p* < 0.001, 95% CI [0.14, 0.34]). This positive association is similarly corroborated in H_2b_, referring to team performance, but only at Time 1 (β = 0.12, *p* = 0.034, 95% CI [0.03, 0.22]).

In this line and concerning H_3_, the results show that IR positively predicted the athletes’ team adherence intention (β = 0.16, *p* < 0.01, 95% CI [0.07, 0.26]). Concerning the association between individual (H_4a_) and team (H_4b_) performance at Time 2, and team adherence intention the following season, the results showed a positive association only for team performance (β = 0.43, *p* < 0.001, 95% CI [0.25, 0.61]).

Finally, regarding H_5_, IR showed a positive and indirect association between coach need-supportive style and individual performance in both time points (Time 1: β = 0.15, *p* < 0.001, 95% CI [0.07, 0.25]; Time 2: β = 0.13, *p* = 0.001, 95% CI [0.06, 0.15]). In addition, IR played an indirect positive role between coach need-supporting and team performance at Time 1 (β = 0.07, *p* = 0.048, 95% CI [0.01, 0.14]). Lastly, IR was only a positive mediator between coach need-supporting and team adherence intention the following season (β = 0.09, *p* = 0.014, 95% CI [0.03, 0.17]).

## Discussion

The present study analyzed the relationship between interpersonal coaching styles, […], and team adherence intention the following season. As shown in the results section, a positive and direct relationship between coach need-supportive style and IR (H_1a_) was confirmed. These results are consistent with previous studies such as that of Fletcher and Sarkar [13], who revealed that numerous psychological factors, including perceived social support, protected athletes from the negative effects of stressors. However, in this study, as in several previous investigations [28, 30, 54], the participants were elite athletes. Therefore, some comparisons can be drawn between the positive results obtained in the present work. H_1_ is partially confirmed.

In addition, the positive and direct relationship between IR and perceived individual (H_2a_) and team (H_2b_) performance at Time 1 is noteworthy. However, at Time 2, this relationship only manifests individual performance. The lack of a relationship between IR and team performance at Time 2 may be because the team did not have a goal to strive for at the end of the season. That is, they were no longer in a stressful (positive or negative) situation before the next game. Thus, applying different strategies to improve resilience progressively and adaptively over time leads to the athlete’s positive evolution in various abilities, including sports performance [55]. However, in developing resilience, three areas need to be considered: personal qualities, an enabling environment, and a challenge mentality, all of which must be addressed to improve the athletes’ ability to withstand pressure [55]. In this regard, it is not only necessary to have a supportive figure in the coach but to extend this figure to several areas considering different opportunities and contexts.

Furthermore, at the team level, the way individuals’ collective qualities (e.g., roles, commitment, support) enable each team player to develop and perform better in the sporting context is important [56, 57]. Thus, it should be noted that when promoting resilience, the focus should be on individual capacity-building and interpersonal relationships, shared processes and group functioning [55]. Even in the study by Codonhato et al. [12], where performance was not the main focus, the gymnasts with higher levels of resilience could maintain optimal performance after recovering from an injury. Therefore, the results of this research partially confirm H_2_, in agreement with previous studies [58–60].

The results showed a positive and direct relationship between IR and team adherence intention the following season (H3). As mentioned in the theoretical framework, no previous studies in the sports field corroborated these results. This may be because when athletes perceive that they are achieving their goals and overcoming adversity on a team that complements them as athletes, they wish to continue to be part of a team that meets their athletic needs. This relationship has been studied in other fields, and the results align with those obtained in this study [25–27]. This is an incipient avenue of research in sports training, where developing and carrying out appropriate strategies for building IR will positively affect adherence to sports practice. The results of this study confirm H_3_.

Regarding H_4_, the results of the present study showed a direct and positive relationship between team performance perceived by the players at Time 2 (H_4b_) and team adherence intention. It is noteworthy that this is the age group with the highest number of sports dropouts, which is why the development of competence associated with performance will influence athletes’ adherence [61]. Previous studies, such as that of Fierro-Suero et al. [62], have confirmed the direct relationship between performance and persistence in sports. Therefore, young people’s perceived performance predicts their intention to remain active. This relationship may be because perceived team performance may affect self-esteem, self-belief, and the ability to excel [63]. The figure of the coach in this perception of performance is essential because if the coach acknowledges the athlete’s effort, provides adequate feedback, and supports players’ achieving their sporting objectives, this will guarantee the future intention to continue practicing the sport on the same team [54]. This is why the results of the present study partially confirm H_4_. Consequently, it is necessary to establish strategies that promote individual perceived competence to predict this team adherence intention.

One of the important findings of this work is the indirect role of IR in the relationships between coach need-support and individual (Time 1 and Time 2; H_5a_) and team (Time 1; H_5b_) performance, and intention to remain on the team (H_5c_). Recent studies have confirmed the direct relationship between coach’s influence and individual and team performance attainment [64–66] and team adherence intention [67–69]. Therefore, given the results of the present study, IR was shown to be a positive and indirect variable, partially supporting H_5_.

### Limitations and future directions

One of the limitations of our work is that the sample between boys and girls is not balanced, only partially representing these young players. Future works should include more female players to analyze possible gender differences. Furthermore, due to the small number of teams and players in some teams, we could not control the possible effect of the team on the relationships of the hypothetical model. Therefore, future studies should include more teams and players (nested into teams) to analyze the associations between the variables included in this study in a multilevel analysis. Another limitation is the lack of measurements in the first half of the season. Future works should include more measurements to understand the evolution of the variables and their relationship throughout a whole season. It would also be interesting to analyze the participants’ IR using the total resilience score to determine different profiles of resilient players. Finally, given that no experimental and/or quasi-experimental research was performed, we could not assess the cause and effect of these relationships. Therefore, it would be advisable to carry out an intervention program to analyze the cause-effect of the variables studied for future work.

Finally, although there is flourishing research on psychological resilience in elite athletes [22, 70], there is little empirical research on sports in training categories. Characteristics of the sports environment, such as interpersonal relationships and positive coach behaviors that support athletes during tasks, enable them to develop resilience and life skills, self-efficacy, and self-esteem [19]. Thus, these characteristics are positive for this stage of training.

However, these environments can be stressful, as they are characterized by achievement targets and pressures, with uncertain chances of success [71]. In addition, factors such as organizational stress related to the athletes’ educational stage, training and the competition itself can also affect psychological well-being [72]. Therefore, programs should be implemented to improve physical (in terms of performance), psychological (in terms of resilience and mental health), and social (in terms of their team adherence intention) well-being. In other words, sports professionals should remember that they should not only train their athletes at a physical/technical level but also a psychological level and, above all, be very conscientious because poor coach-athlete interactions can lead to a loss of self-evaluation [73].

## Conclusion

In conclusion, it is necessary to highlight the importance of the coaches’ interpersonal style in athletes’development of IR, favoring a supportive attitude. Consequently, it would be ideal to promote and develop strategies to build this skill during training and/or competitions. As a consequence of improving IR, players’ perceived individual and team performance may both increase. The combination of these factors is fundamental for developing team adherence intention the following season and promoting adherence to sports practice in young people.

For this reason, IR may be an antecedent of positive consequences and act as a facilitator between the coach’s interpersonal style and the athletes’ sports performance, as well as their future team adherence intention. In this regard, sports professionals should emphasize that feeling vulnerable to stress or having difficulty coping with adversity should not be perceived as weaknesses. On the contrary, they may be a sign of strength and a starting point of positive change that would help resist and thrive in high-pressure situations. Therefore, it is recommended to work on strategies that enable athletes to cope with positive and negative stress situations. The results of the present work support the idea that youth sports may be a suitable avenue for developing resilience and have implications for the future practice of sports coaching.

## Data Availability

The data presented in this study are available on request from the corresponding author.
